# Making sense of mental pain in adolescents in the era of social media

**DOI:** 10.1192/j.eurpsy.2026.10302

**Published:** 2026-07-31

**Authors:** M. Pompili

**Affiliations:** Sapienza University of Rome, Rome, Italy

## Abstract

**Abstract:**

Classical suicidology conceptualizes suicide as an attempt to escape from unbearable psychological pain (*psychache*), rather than as a movement toward death. Suicide emerges when emotional suffering becomes intolerable and consciousness itself is experienced as painful, driven by persistent negative emotions and self-referential internal dialogue. In such conditions, individuals may perceive the cessation of consciousness as the only viable relief from anguish.

Beyond intrapsychic distress, childhood maltreatment represents a major vulnerability factor for suicidal behavior, contributing to long-term susceptibility to psychological pain. In contemporary societies, particularly among adolescents and young adults, these established risk factors may be further influenced by the pervasive role of social media. Digital environments can intensify psychological suffering through social comparison, cyberbullying, exposure to self-harm–related content, and reinforcement of negative self-concepts, while simultaneously limiting opportunities for emotional regulation and interpersonal containment.

This presentation examines suicide risk from theoretical and empirical perspectives, focusing on the relationship between psychological pain, childhood trauma, and suicidal behavior, with specific attention to younger populations exposed to online social contexts. Emerging evidence suggests that social media may act as a contextual amplifier of psychological pain in vulnerable youth, particularly in those with prior trauma or affective dysregulation, while also representing a potential avenue for early identification and prevention. An empathic, developmentally informed understanding of suffering remains essential for suicide risk reduction, underscoring the clinician’s role in recognizing and modulating psychological pain within both offline and digital environments.

**Disclosure of Interest:**

M. Pompili Consultant of: M Pompili wishes to disclose that in the last 5 years, he has received lectures and advisory board honoraria or has engaged in clinical trial activities with Angelini Pharma, Allergan, Johnson&Johnson, Lundbeck, Merck Sharp and Dohme, Otsuka, Rovi, Pfizer Inc, Fidia, Viatris, Recordati, Newron, Neopharmed Gentili, EG Stada, Boehringer Ingelheim and Teva, all of which are unrelated to this presentation.

